# Advanced strategies for enhancing kaolin ceramics using nanostructured additives: A comprehensive study

**DOI:** 10.1371/journal.pone.0324449

**Published:** 2025-05-27

**Authors:** Nasser A. M. Barakat, Neama A. M. Eltohamy, Kasem R. M. Abdelrazek, Wesam Elhawam, Ahmed A. A. Ammar, Hassan Fouad, Mohamed Hashem, Hak Yong Kim

**Affiliations:** 1 Chemical Engineering Department, Faculty of Engineering, Minia University, Minia, Egypt; 2 Department of Holographic Expression, Faculty of Art Education, Minia University, Minia, Egypt; 3 Department of Holographic Expression, Faculty of Art Education, Helwan University, Cairo, Egypt; 4 Biomedical Engineering Dept. Faculty of Engineering, Helwan University, Helwan, Egypt; 5 Department of Dental Health, College of Applied Medical Sciences, King Saud University, Riyadh, Saudi Arabia; 6 Department of Nano Convergence Engineering, Jeonbuk National University, Jeonju, South Korea; 7 Department of Organic Materials and Fiber Engineering, Jeonbuk National University, Jeonju, South Korea; University of Szeged, HUNGARY

## Abstract

In this study, we investigated the enhancement of Egyptian kaolin ceramics using various nanostructured additives: polyvinyl alcohol (PVA), titanium dioxide (TiO_2_) nanofibers, carbon nanotubes (CNTs), silicon dioxide (SiO_2_) nanoparticles, and activated carbon (AC). The results showed that PVA and TiO_2_ nanofibers significantly increased the compression strength of the ceramics, with TiO_2_ nanofibers providing the highest improvement at 2.47 MPa. SEM analysis revealed that these additives facilitated better particle agglomeration, leading to improved mechanical properties. TGA indicated a shift in the sintering reaction peak from 511°C for pristine kaolin to 548°C for PVA-modified kaolin and slightly higher for TiO_2_-modified kaolin, suggesting enhanced sintering processes. Additionally, the thermal cycling tests demonstrated that TiO_2_ nanofibers-modified kaolin exhibited exceptional stability, with no change in apparent density, highlighting its potential for refractory applications. PVA-modified samples also showed a significant decrease in porosity and water absorption. These findings underscore the potential of specific nanostructured additives to enhance the mechanical and thermal properties of kaolin ceramics, offering valuable insights for future research and industrial applications.

## 1. Introduction

Kaolin-based ceramics are widely used in industrial applications due to their cost-effectiveness and thermal resistance. However, traditional kaolin ceramics often suffer from poor mechanical strength and high porosity, limiting their performance under mechanical or thermal stress [1,2]. Previous efforts to enhance ceramic materials have included the use of various binders and reinforcing materials, but challenges such as weak particle dispersion, phase instability, and limited scalability remain unresolved [3]. Additionally, most studies focus on a single type of additive without exploring synergistic effects or structural integration [4]. Kaolin, a hydrated aluminum silicate predominantly composed of the mineral kaolinite, stands as one of the most significant industrial clay minerals globally. Its physical and chemical properties render it versatile for a multitude of applications [5,6]. Among these, the ceramics industry emerges as a primary domain, encompassing a diverse array of products ranging from dinnerware to refractories [7,8].

In Egypt, where kaolin holds substantial industrial importance, a reliance on imported kaolin persists despite local deposits meeting approximately 73% of industry demands. However, the disparity lies in the failure of local kaolin to consistently meet required specifications for ceramic production, leading to a significant production of waste materials due to non-compliance [9,10].

Prior studies on Egyptian clays have predominantly focused on mineralogical and chemical compositions, rheology, drying and firing characteristics, and whiteness [11,12]. Notably, high plasticity during shaping, alongside the necessity for high densification temperatures, poses substantial challenges to widespread application [13].

Traditionally, the incorporation of ball clay has been the primary strategy to enhance plasticity, albeit with limitations. However, leveraging the unique properties of nanomaterials might present a promising avenue for improving the characteristics of Egyptian kaolin. In this context, our study explores novel strategies aimed at enhancing the properties of Egyptian kaolin. Interest in the properties of nanomaterials has been growing, driven by their potential to enhance the development of ceramics with improved characteristics [14,15]. A considerable amount of research has explored incorporating nanoparticles (NPs) like Al_2_O_3_ [16,17], ZrO_2_ [18,19], TiO_2_ [20,21], and SiO_2_ [22,23] into ceramic matrices to boost their mechanical strength, thermal stability, and overall suitability for advanced ceramic applications. These NPs can be integrated into ceramic bodies through various techniques, such as airbrush coating on green or fired specimens or by directly adding them as raw materials [14,22,24,25]. Beside the nanomaterials, other materials have been also investigated including egg sell and ammonium acetate [26,27], and sewage sludge and blast furnace slag [28–31].

Specifically, we investigate the efficacy of employing poly (vinyl alcohol) to improve plasticity, alongside the addition of various nanostructures such as functionalized carbon nanotubes, titanium oxide nanofibers, silica nanoparticles, and modified activated carbon to enhance apparent density, compression strength, water absorption, and surface porosity. Notably, these improvements open up new possibilities for the utilization of kaolin in ceramic membranes technology.

The selection of additives is deliberate, allowing us to discern the influence of additive nature (carbonaceous or metal oxides) and nanostructure morphology (long and short axial ratios) on the resultant characteristics of kaolin products. Our findings underscore the profound impact of both morphology and chemical composition of additives on enhancing the properties of Egyptian kaolin, paving the way for advanced ceramic applications. Through this manuscript, we aim to contribute to the ongoing discourse on optimizing industrial clay minerals, particularly Egyptian kaolin, for enhanced utility in ceramic production, thereby fostering innovation and sustainability in materials engineering.

## 2. Experimental work

### 2.1 Materials

Egyptian kaolin has been obtained from the local market in Aswan city. Table 1 shows the chemical composition of the used kaolin. The kaolin was crushed, grinded and sieved to pass the 200 ASTM mesh sieve.

**Table 1:** Chemical composition (%) of the Egyptian clay [9].

SiO_2_	TiO_2_	Al_2_O_3_	Fe_2_O_3_	MnO	MgO	CaO	Na_2_O	K_2_O	P_2_O_5_
45-50	1.5-2.8	32-38	0.7-0.9	0.0-01	0.0-0.01	0.0-0.2	0.0-0.05	0.0-0.1	0.0-0.1

Poly (vinyl alcohol) (PVA, M.wt.79000, Alfa Aesar), Polyvinylpyrrolidone (PVP, 90K, Sigma Aldrich) and titanium isopropoxide (TTIP, Ti(OCH(CH_3_)_2_)_4_, Sigma Aldrich) were used as received. Multiwall carbon nanotubes (CNTs) were purchased from nanotechnology lab in Beni-Swef university, Egypt). Activated carbon was obtained from Eisen-Golden Laboratories. Glacial acetic acid and dehydrated ethanol were used in the preparation of TiO_2_ nanofibers.

### 2.2 Fabrication of TiO_2_ nanofibers

Titanium oxide nanofibers were prepared by electrospinning process. The electrospun solution was prepared by adding 1 g titanium isopropoxide to a solution containing 2 g glacial acetic acid and 2 g un-hydrated ethanol followed by stirring for 15 min. Later, 6 g ethanol and 1 g PVP were added into the prepared solution and stirred until getting transparent sol-gel. The electrospinning process was performed at 12 kV DC power in a standard electrospinning setup. The tip-to-collector distance was kept at ~ 15 cm. The collected nanofiber mats were vacuously dried at 60 °C for 24 hours. Finally, the dried mats were calcined under air at 600 °C for 2 h with a heating rate of 2 deg/min.

### 2.3 Silicon oxide nanoparticles

The process of synthesizing silica nanoparticles from rice husk involves several steps, starting with the rinsing of RH in alcohol and deionized water to remove impurities and contaminants. Subsequently, the dried RH is crushed, ground, and sieved to achieve uniform particle size (70–75 µm), ensuring homogeneity in the resulting silica nanoparticles. Calcination of the prepared RH under air at 700°C, with a heating rate of 3°C/min and a 2-hour holding time, leads to the decomposition of organic components present in RH, such as cellulose and lignin, and the conversion of silica precursors into amorphous silica nanoparticles.

### 2.4 Functionalization of carbonaceous materials

The used activated carbon and carbon nanotubes have hydrophobic property. Therefore, to get homogeneous water slurry from these materials, functionalization process was performed by making a reflux treatment using sulfuric acid/nitric acid solution. In a clean and dry glass beaker, the required quantities of sulfuric acid and nitric acid in a ratio of 3:1 by mass were measured. The measured acids were added to the beaker, ensuring proper ventilation in a fume hood. The acid mixture was gently stirred using a glass stirring rod to ensure homogeneity. The desired quantity of activated carbon or carbon nanotubes was measured and transferred into a clean round-bottom flask. The prepared acid solution to the flask containing the carbonaceous materials to have a 1 wt% slurry. The round-bottom flask equipped with a reflux condenser is placed onto a magnetic stirrer. The reflux apparatus was assembled carefully to ensure a tight seal between the flask and the condenser to prevent vapor escape. The reaction mixture was heated under reflux conditions to keep boiling for 1 hour. After the reflux treatment, the reaction mixture was allowed to cool to room temperature naturally. Once cooled, the contents of each flask into were transferred to separate clean glass beakers. The acid-treated activated carbon and carbon nanotubes were diluted with distilled water to neutralize the acidity. The suspension was filtered using a vacuum filtration setup with a filter paper. The acid-treated carbonaceous materials were washed several times with distilled water to remove any residual acid. After washing, the produced materials were dried under vacuum at 60 °C for one night. Table 2 summarizes the used additives.

**Table 2:** Summary of nanostructured additives used in this study, including type, morphology, dimensions, and source or preparation method.

Additive	Type	Morphology	Size/Dimension	Source/Preparation
**PVA**	Organic polymer	Granules	~	Commercial (Sigma Aldrich)
**TiO**_**2**_ **NFs**	Inorganic metal oxide	Nanofibers	~ 200 nm diameter	Synthesized via electrospinning
**CNTs**	Carbon nanostructure	Nanotubes	20 ~ 40 nm diameter	Treated (acid functionalized)
**SiO**_**2**_ **NPs**	Metal oxide nanoparticles	Nanoparticles	~ 50 nm diameter	Prepared via rice husk calcination
**AC**	Amorphous carbon	Micro particles	< 75 µm	Treated (acid functionalized)

### 2.5 Experimental procedure

#### 2.5.1 Samples preparation.

First, slurries from the proposed additives were prepared by adding 0.08 g of each individual additive to 45 mL of distilled water, followed by ultrasonication for 30 minutes to ensure complete dispersion. 150 g of Kaolin powder was wetted by 45 mL of different aqueous solutions to prepare the kaolin mud for all samples. For the first group of samples (coded by -1), the utilized aqueous solution consists of 10 mL of the prepared additive slurry mixed with 35 mL of PVA aqueous solution containing 1 g PVA. For the second group of samples (coded by -2), 15 mL of the additive slurry was mixed with 30 mL of PVA aqueous solution containing 1 g PVA to prepare the required 45 mL aqueous solution for kaolin mud preparation. While in the third group of samples (coded by -3), 20 mL of the additive slurry was mixed with 25 mL of PVA aqueous solution containing 1 g PVA. PVA was used only (PVA sample) by dissolving 1 g of the polymer in 45 ml. The mud mixture was thoroughly mixed with the prepared additive/PVA solutions until homogeneity was achieved.

#### 2.5.2 Cube samples formation.

The homogenized kaolin muds were molded into cubes with dimensions of 2 × 2 × 2 cm³. The formed cubes were dried in an oven at 100°C for 24 hours to remove any residual moisture. Subsequently, two series of samples were prepared: one was calcined at 900 °C and the other at 1050 °C, in order to evaluate the influence of sintering temperature on the physical and mechanical properties of the produced ceramic materials. Then, the cubes became ready for the characterization process. The experimental procedure was replicated for each group of samples to ensure consistency and reliability of results. Control samples without additives were also prepared following the same procedure for comparison purposes.

### 2.6 Characterizations

The produced materials underwent comprehensive characterization at the Central Laboratory for Microanalysis and Nanotechnology, Minia University. The morphological features of the materials were investigated using scanning electron microscopy (SEM) with a Hitachi S-7400 scanning electron microscope from Japan. For comprehensive analysis of the chemical composition, X-ray diffraction (XRD) was performed using a Rigaku X-ray diffractometer (Tokyo, Japan) equipped with a Cu Kα radiation source (λ = 1.5406 Å). The test conditions included a voltage of 40 kV and a current of 30 mA. The samples were scanned over a 2θ range of 5° to 90° with a step size of 0.02° and a counting time of 1 second per step. Additionally, transmission electron microscopy (TEM) analysis was conducted utilizing a Phillips CM12 TEM instrument equipped with an elemental mapping tool. To prepare the samples for TEM analysis, a copper grid was immersed in the sample slurry and air-dried prior to loading into the instrument. Before loading, the samples underwent sonication in pure ethanol for 10 minutes to ensure uniform dispersion and deposition onto the grid.

To measure the apparent density, water absorption and the surface porosity, the pristine and modified of clay cubes, the weight of the dry sample (*W*_*D*_) was estimated then the cube was soaked in water for 30 min, then the weight of the soaked cubes (*W*_*S*_) has been estimated. After removing from water, the excess water around the cubes has been removed by cleaning tissue, then the weight of the cleaned cubes (*W*_*C*_) was estimated. Later, the following equations were used to estimate the properties:


$$\begin{array}{c} Apparent\;density=\;\frac{W_{D}}{W_{C}-W_{S}}\;g/{cm}^{3} \end{array}$$ (1)

$$\begin{array}{c} Water\;absorption=\;\frac{W_{C}-W_{D}}{W_{D}}\times 100\; \end{array}$$ (2)

$$\begin{array}{c} Apparent\;porosity=\;\frac{W_{C}-W_{D}}{W_{C}-W_{S}}\times 100 \end{array}$$ (3)

The plasticity was measured by shaping rods from various mud samples formed by adding different amounts of water (or additive slurry) to the kaolin powder, the rod diameter was kept at 3.25 mm. Water (or slurry) content changes until appearing cracks on the rod. Then, the rod was subjected to drying at 110 °C for 24 h to estimate the exact weight of water in the cracked rod. Then the plasticity is calculated from this equation [32]:


$$\begin{array}{c} Water\;absorption=\;\frac{Exact\;water\;weight\;in\;the\;cracked\;rod}{Weight\;of\;the\;dry\;rod}\times 100\; \end{array}$$ (4)

The experiment has been repeated three times and the average value was used.

ASTM C109 compression test has been achieved to investigate the mechanical properties using Tinius Olsen (H5KT model) Benchtop Tester (USA).

## 3. Results and discussion

### 3.1 Additives characteristics

#### 3.1.1 Titanium oxide nanofibers.

**Fig 1:**
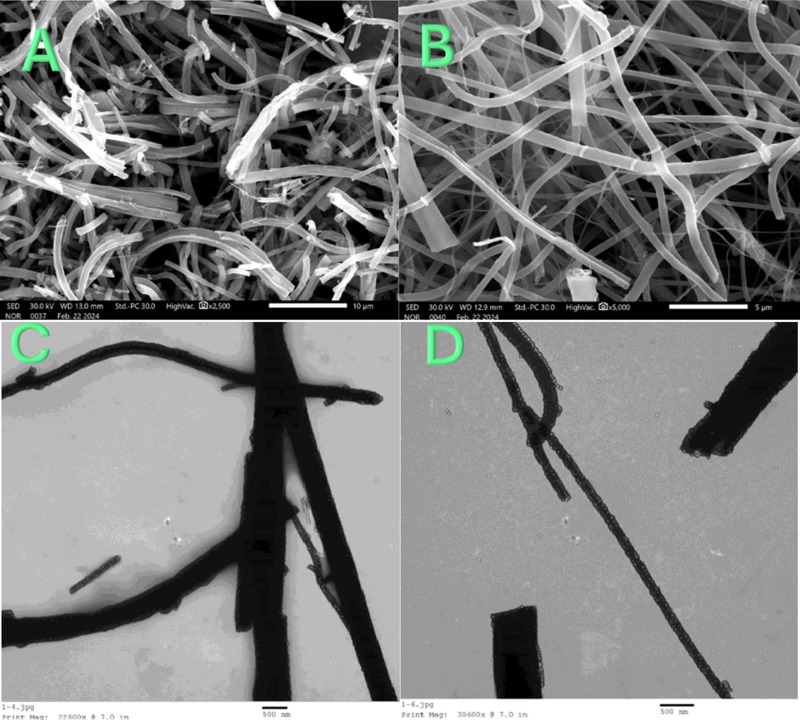
Two magnifications SEM; (A and B) and TEM (C and D) images for the produced TiO_2_ nanofibers.

The SEM images displayed in Fig 1A and 1B depict the exceptional morphological characteristics of the synthesized titanium oxide nanofibers, which were produced via the electrospinning process of a sol-gel solution comprising titanium isopropoxide, polyvinylpyrrolidone (PVP), anhydrous ethanol, and glacial acetic acid. The electrospun mats were subsequently subjected to vacuum drying at 60°C followed by calcination in air at 650°C. The observed smooth, long axial ratio nanofibers devoid of bead-like structures signify a high level of uniformity and integrity in the nanofibrous morphology.

The utilization of titanium isopropoxide as the precursor in the sol-gel solution plays a crucial role in determining the final morphology of the nanofibers. Titanium isopropoxide undergoes hydrolysis and polycondensation reactions during the sol-gel process, leading to the formation of titanium oxide nanofibers. The addition of PVP, a commonly used polymer in electrospinning, aids in the formation of a stable electrospinnable solution and assists in the alignment and stretching of the nanofibers during the electrospinning process[33,34].

Anhydrous ethanol serves as a solvent in the sol-gel solution, facilitating the dissolution of titanium isopropoxide and PVP and ensuring homogeneous mixing of the components. Glacial acetic acid, acting as a catalyst, promotes the hydrolysis of titanium isopropoxide and controls the rate of gel formation, thereby influencing the final morphology of the electrospun nanofibers [35]. The vacuum drying process at 60°C helps remove residual solvent and moisture from the electrospun mats, ensuring proper consolidation of the nanofibrous structure. Subsequent calcination in air at 650°C induces the decomposition of organic components, such as PVP, and the conversion of amorphous titanium oxide precursors into crystalline titanium oxide nanofibers. The controlled heating conditions during calcination play a crucial role in preserving the integrity of the nanofibrous morphology while promoting phase transformation and crystallization [36].

The absence of bead-like structures and the presence of smooth, continuous nanofibers can be attributed to the synergistic effects of precursor chemistry, polymer matrix, solvent selection, and processing parameters employed during electrospinning and subsequent thermal treatment. The successful synthesis of uniform titanium oxide nanofibers with desirable morphological characteristics underscores the importance of sol-gel chemistry and electrospinning techniques in tailoring the properties of nanomaterials for various applications.

The TEM images presented in Fig 1C and 1D provide further insights into the morphology and structural characteristics of the synthesized nanofibers. The images reveal distinct black nanofibers, indicative of high crystallinity in the produced material. This observation aligns with the expected crystalline nature of titanium oxide nanofibers and will be further corroborated by X-ray diffraction (XRD) analysis. The black appearance of the nanofibers in the TEM images suggests that the material possesses a high degree of electron density, characteristic of crystalline structures. The contrast between the dark nanofibers and the surrounding background indicates variations in electron scattering within the sample, which can be attributed to differences in material composition, thickness, and crystalline arrangement [37,38].

The high crystallinity observed in TEM Images is consistent with the anticipated transformation of amorphous titanium oxide precursors into crystalline nanofibers during the calcination process. The controlled heating conditions during calcination promote the removal of organic components and the crystallization of titanium oxide, resulting in the formation of well-defined crystalline structures.

**Fig 2:**
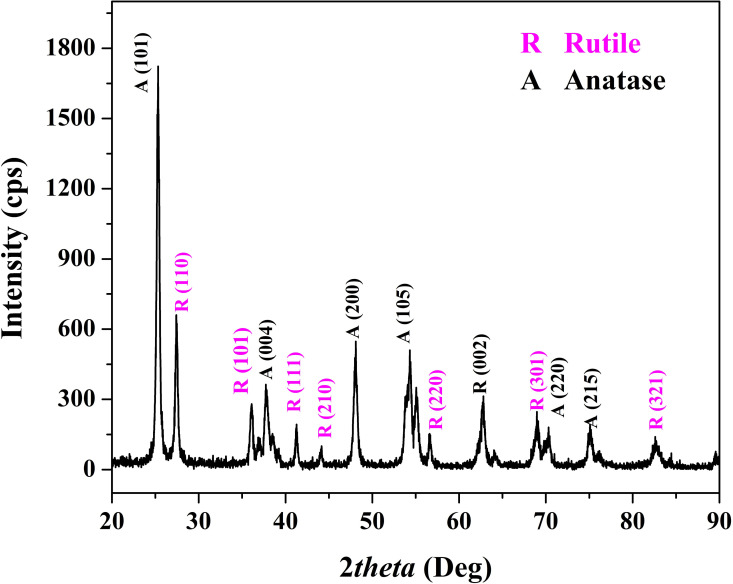
XRD pattern for the prepared titanium oxide nanofibers.

The X-ray diffraction (XRD) analysis of the prepared TiO_2_ nanofibers, Fig 2, revealed the presence of both anatase and rutile phases. This mixed-phase composition is significant as it can influence the mechanical and functional properties of the ceramic materials.

The anatase phase of TiO_2_ is characterized by several prominent diffraction peaks. According to the International Centre for Diffraction Data (ICDD) database, the standard card number for anatase TiO2 is 21–1272. The most prominent peaks for anatase TiO_2_ occur at the following 2θ angles of 25.3°, 36.9°, 37.8°, 48.1°, 53.4°, 55.1°, 62.7° and 75.0° corresponding to (101), (103), (004), (220), (105), (211), (204) and (215), respectively. These peaks are indicative of the tetragonal crystal structure of anatase, which is known for its high photocatalytic activity and surface area.

The rutile phase of TiO_2_ is another stable crystalline form and has distinct diffraction peaks. The standard card number for rutile TiO_2_ in the ICDD database is 21–1276. The primary peaks for rutile TiO_2_ appear at the following 2θ angles 27.4°, 36.1°, 41.3°, 44.1°, 54.3°, 56.6°, 62.7° and 69.1° corresponding to (110), (101), (111), (211), (220), (002), (301) and (112) crystal plans, respectively. The rutile phase has a denser, more stable tetragonal structure compared to anatase and contributes to enhanced mechanical strength and thermal stability of the ceramics.

Formation of titanium oxide is expected as a normal route for thermal decomposition of the used precursor; titanium isopropoxide. The presence of both anatase and rutile phases in the TiO_2_ nanofibers suggests a synergistic effect that can be advantageous for the overall performance of the modified kaolin ceramics. The anatase phase contributes to improved surface properties, which can enhance the interaction between the nanofibers and the kaolin matrix. This interaction is crucial for achieving better dispersion and uniformity within the ceramic body, leading to improved mechanical properties such as compression strength. On the other hand, the rutile phase adds to the thermal stability and structural integrity of the nanofibers. The higher density and stability of the rutile phase can provide resistance to high-temperature deformation and contribute to the overall durability of the ceramic material. The combination of anatase and rutile phases in the TiO_2_ nanofibers likely facilitates a balance between enhancing surface interactions and maintaining structural stability [39,40].

#### 3.1.2 Silicon oxide nanoparticles.

**Fig 3:**
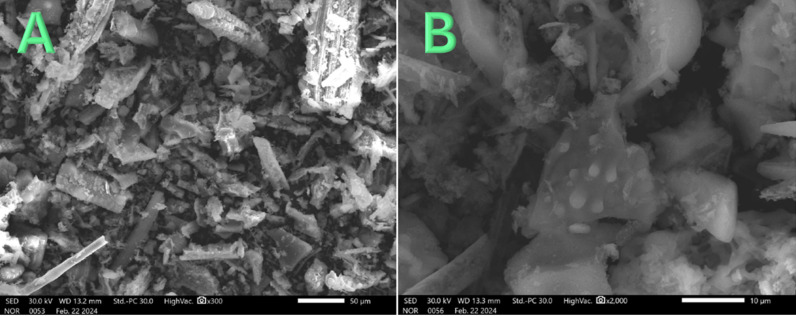
Two magnifications SEM images for the produced SiO_2_ nanoparticles.

The SEM images displayed in Fig 3 showcase the morphology of the synthesized silicon oxide (silica) nanoparticles obtained from the calcination of rice husk (RH). The images reveal nanoparticles with a random morphology and a wide range of sizes, indicating the successful transformation of RH into silica nanoparticles through the calcination process [41]. The utilization of rice husk as a precursor for silica nanoparticles offers several advantages, making it an attractive an`d sustainable option for nanoparticle synthesis. Rice husk, an agricultural waste product, is abundantly available and renewable, making it a cost-effective and environmentally friendly precursor for silica nanoparticles synthesis. The controlled calcination conditions are crucial for achieving desired properties and morphology in the synthesized silica nanoparticles [42].

The random morphology observed in the SEM images reflects the heterogeneous nature of the precursor material and the complex structure of rice husk. The varying sizes of the nanoparticles can be attributed to differences in the distribution and composition of silica precursors within the RH matrix, as well as the calcination process parameters [43]. Furthermore, the synthesis of silica nanoparticles from rice husk offers potential applications in various fields, including catalysis, drug delivery, sensors, and environmental remediation. The unique properties of silica nanoparticles, such as high surface area, tunable porosity, and biocompatibility, make them promising candidates for diverse applications.

**Fig 4:**
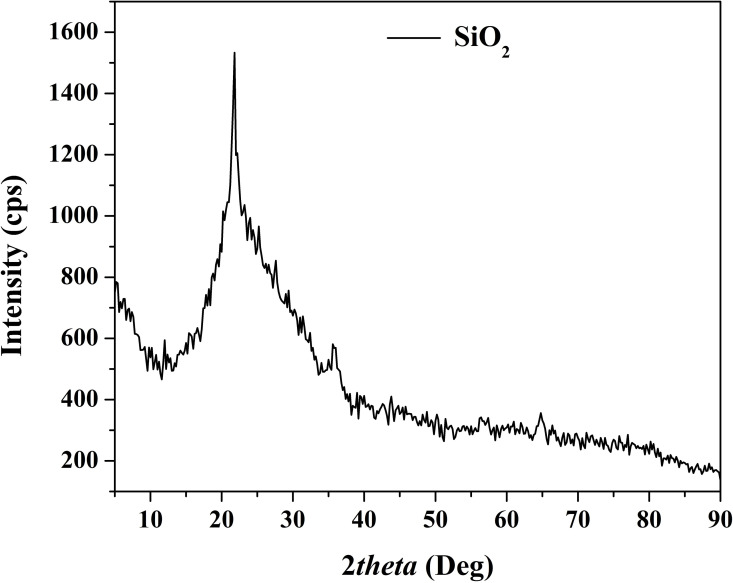
XRD pattern for the prepared silicon oxide nanoparticles.

The X-ray diffraction (XRD) pattern presented in Fig 4 reveals distinctive peaks at specific 2θ values, which are indicative of the crystallographic structure of the produced silicon dioxide (SiO_2_) nanoparticles. However, unlike titanium dioxide (TiO_2_), which typically exhibits sharp and high-density peaks in the XRD pattern, SiO_2_ shows wider and comparatively lower intensity peaks. This observation suggests that the synthesized SiO_2_ nanoparticles may possess an amorphous or partially crystalline structure.

The broadening and reduced Intensity of the XRD peaks in the SiO_2_ pattern can be attributed to several factors, including the amorphous nature of the nanoparticles and the presence of structural defects or disorder. Amorphous materials lack long-range order in their atomic arrangement, resulting in diffuse scattering of X-rays and broader peaks in the XRD pattern. Additionally, the presence of crystalline imperfections or grain boundaries can contribute to peak broadening and reduced intensity. Another possible reason for the observed XRD pattern characteristics could be the presence of nanoscale crystallites or nanodomains within the SiO_2_ nanoparticles. Nanosized crystallites can lead to peak broadening due to size effects, such as strain and finite size contributions, resulting in lower intensity and wider peaks in the XRD pattern [44]. The observed peaks match the standard peaks of Cristobalite SiO_2_ according to the ICDD reference pattern PDF# 27–0605.

The synthesis conditions and processing parameters employed during the preparation of SiO_2_ nanoparticles can also influence their crystallinity and XRD pattern characteristics. For instance, variations in calcination temperature, heating rate, and duration can impact the degree of crystallinity and structural ordering in the synthesized nanoparticles [45].

### 3.2 Characterizations of the modified kaolin

In the applied characterizations, all data points in the following figures represent the average of three independent measurements, with standard deviations consistently below ±3%. Experimental trends were reproducible across replicates.

#### 3.2.1 Plasticity limit.

**Fig 5:**
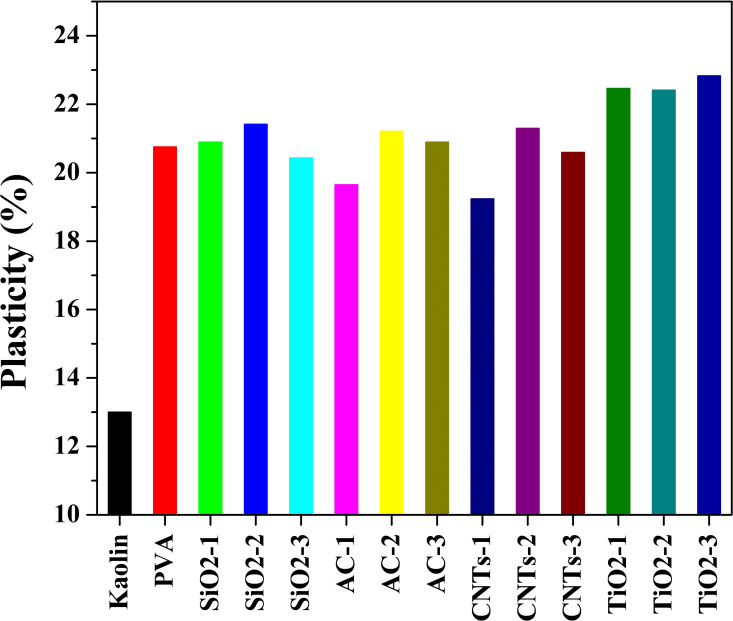
Plasticity limit (%) of pristine and modified Egyptian kaolin.

The plasticity of kaolin mud is a crucial factor in its shaping process, as it determines the ability of the mud to be molded into desired forms without cracking or deforming. Low plasticity limit in Egyptian kaolin mud has been identified as a significant challenge in ceramic production. Fig 5 illustrates the plasticity enhancement achieved by incorporating proposed additives into the kaolin mud, addressing this issue.

The measured plasticity of pristine kaolin mud was found to be 13.122%. However, the utilization of proposed additives led to notable improvements in plasticity, with values ranging from 19.2368% to 22.8284%. These results signify the effectiveness of the additives in enhancing the plasticity of the kaolin mud, thereby improving its suitability for shaping and forming processes in ceramic production. The plasticity enhancement achieved in kaolin mud with the incorporation of SiO_2_, TiO_2_, activated carbon (AC), and carbon nanotubes (CNTs), along with polyvinyl alcohol (PVA), was investigated across three different concentrations (low; code -1, medium; code -2, and high; code -3). The significant findings and underlying mechanisms associated with each additive and concentration level is discussed below:

1. *Polyvinyl alcohol* (PVA): PVA, utilized in conjunction with all additives, acts as a binder and plasticizer, enhancing the interparticle interactions and facilitating better dispersion of other additives within the kaolin matrix. The addition of PVA contributes to increased plasticity by promoting better cohesion and fluidity of the kaolin mud, leading to improved shaping and forming characteristics.
2. *Silica nanoparticles*: SiO_2_ nanoparticles, when combined with PVA, effectively improve plasticity by acting as lubricants between kaolin particles. At low concentrations (SiO_2_-1), the lubricating effect of SiO_2_ nanoparticles is moderate, resulting in a modest increase in plasticity. Medium (SiO_2_-2) concentration of SiO_2_ nanoparticles further enhance plasticity by reducing friction and facilitating smoother particle movement within the mud matrix. However, more increase in the concentration (SiO_2_-3) showed relatively negative impact which could be attributed to start formation of barrier layers between the clay particles.
3. *Titanium dioxide nanofibers*: The plasticity enhancement achieved with the incorporation of titanium dioxide (TiO_2_) nanofibers, in conjunction with polyvinyl alcohol (PVA), was investigated across three different concentrations (low, medium, and high). TiO_2_ was utilized in the form of nanofibers possessing a long axial ratio compared to SiO_2_ nanoparticles. The long axial ratio of TiO_2_ nanofibers facilitates better particle dispersion and interaction within the mud matrix, leading to improved plasticity. Accordingly, it is shown that in Fig 5, TiO_2_-containing samples reveal higher plasticity compared to SiO_2_-based samples. Plasticity improvement at the low and moderate concentration samples (TiO_2_-1 and TiO_2_-2) were almost the same. The increase in plasticity observed at these concentration levels suggests that the TiO_2_ nanofibers effectively contribute to reducing interparticle friction and enhancing particle mobility [46]. The enhanced plasticity observed indicates that the TiO_2_ nanofibers effectively promote better particle dispersion and interparticle interactions, resulting in improved flowability and moldability of the kaolin mud. High concentrations of TiO_2_ nanofibers (TiO_2_-3) exhibit the most pronounced improvement in plasticity when combined with PVA. The substantial increase in plasticity at this concentration level suggests that the TiO_2_ nanofibers create a highly lubricating environment, allowing for optimal flow and shaping of the kaolin mud, thus overcoming the limitations associated with low plasticity.
4. *Activated Carbon* (AC): The observed trend in plasticity enhancement with the incorporation of activated carbon (AC) in kaolin mud, in conjunction with polyvinyl alcohol (PVA), reveals interesting insights into the role of AC concentration on plasticity improvement. The results indicate that while a small amount of AC (AC-1 sample) led to a slight decrease in plasticity compared to PVA only, increasing the AC content resulted in significant improvements in plasticity.

The slight decrease in plasticity observed in the AC-1 sample compared to the PVA-only sample may be attributed to the initial interaction between AC particles and the kaolin matrix. The presence of a small amount of AC may slightly disrupt the rheological properties of the kaolin mud, resulting in decreased flowability and moldability.

In contrast, increasing the AC content in the AC-2 and AC-3 samples resulted in notable improvements in plasticity compared to the PVA-only sample. At medium and high concentrations, AC particles are more effectively dispersed within the mud matrix, leading to enhanced particle interaction and lubrication between kaolin particles. The increased surface area and porosity of AC at higher concentrations contribute to better water retention and rheological properties of the mud, thereby improving plasticity. Additionally, the presence of AC may act as a reinforcing agent, enhancing the mechanical stability of the mud matrix and reducing cracking during shaping processes.

These findings highlight the importance of optimizing AC concentration to achieve the desired plasticity enhancement in kaolin-based materials. By carefully adjusting the AC dosage, it is possible to tailor the rheological properties of kaolin mud for specific applications in ceramic production and other industrial sectors.

1. *Carbon Nanotubes* (CNTs): The observed variation in plasticity enhancement with the incorporation of carbon nanotubes (CNTs) in kaolin mud, in conjunction with polyvinyl alcohol (PVA), highlights the complex interplay between CNT concentration and plasticity improvement. Interestingly, while the lowest plasticity was observed at low CNT concentration (CNTs-1), moderate and high CNT contents (CNTs-2 and CNTs-3) resulted in significant improvements in plasticity, approaching or surpassing the plasticity observed with PVA alone.

Limited CNT loading, in the case of CNTs-1 sample, may result in inadequate lubrication and reinforcement effects, leading to reduced flowability and moldability of the kaolin mud. Additionally, CNTs at low concentrations may not effectively contribute to improving water retention or rheological properties of the mud, further impacting plasticity. In contrast, increasing the CNT content in the CNTs-2 and CNTs-3 samples led to notable improvements in plasticity, approaching or exceeding the plasticity observed with PVA alone. At moderate and high concentrations, CNTs are more effectively dispersed and interact with kaolin particles, leading to enhanced lubrication and reinforcement effects. Moreover, the increased loading of CNTs promotes better particle dispersion and alignment within the mud matrix, resulting in improved flowability and moldability.

It is note worth mentioning that, in ceramic manufacturing, particularly in applications such as membrane casting or tile pressing, a plasticity limit above 18% is typically considered suitable for achieving sufficient workability and reducing the risk of cracking during shaping and drying stages. The enhanced plasticity observed in the modified kaolin samples meets or exceeds this threshold, highlighting the potential of most of the used additives for such applications.

#### 3.2.2 Compression strength.

**Fig 6:**
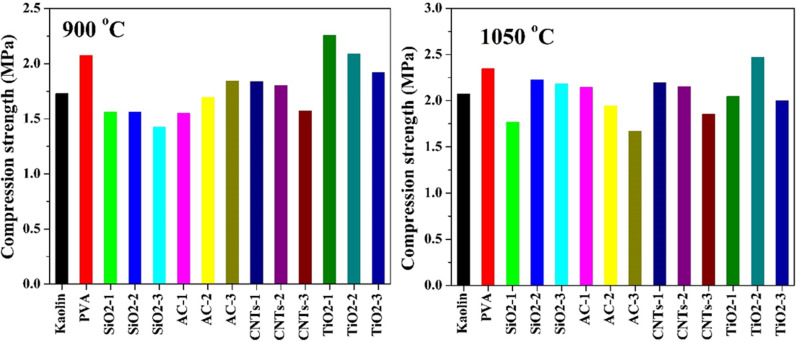
Compression strength of pristine and modified Egyptian kaolin sintered at two temperatures: 900 and 1050 °C.

The compression strength measurements provide valuable insights into the mechanical properties of the pristine and modified kaolin samples, shedding light on the effectiveness of various additives in enhancing the strength characteristics. The samples have been sintered at two temperatures: 900 and 1050 °C. The results are displayed in Fig 6.

3.2.2.1 **At 900 °C:** The observed trends in compression strength, compared to the pristine kaolin and PVA-only samples, reveal the complex interplay between additive type, concentration, and resulting material properties. PVA, when used alone, significantly increases the compression strength of kaolin samples (2.075 MPa) compared to the pristine kaolin (1.7275 MPa). This improvement can be attributed to the binding and strengthening properties of PVA, which enhance the cohesion and integrity of the kaolin matrix. However, different effects of the other additives have been observed.

Incorporating SiO_2_ nanoparticles, in conjunction with PVA, resulted in a decrease in compression strength compared to the pristine kaolin. This reduction may be attributed to the dispersing effect of SiO_2_ nanoparticles, which may compromise the structural integrity of the kaolin matrix at certain concentrations. Numerically for the low, moderate and high concentrations, SiO2-1, SiO2-2 and SiO2-3, the observed compression strength was 1.56, 1.558 and 1.425 MPa, respectively.

At low and moderate concentrations, the compression strength of AC-based samples was lower compared to the pristine kaolin, indicating a potential weakening effect of AC on the kaolin matrix at these concentrations. However, increasing the AC content led to an improvement in compression strength, surpassing that of the pristine kaolin at high concentrations. This improvement may be attributed to the reinforcing effect of AC, which enhances the mechanical stability and integrity of the kaolin matrix.

The compression strength of CNTs-based samples exhibited a gradual decrease with increasing CNT content, suggesting that CNTs may not effectively contribute to enhancing the mechanical properties of the kaolin matrix at the concentrations tested. Although some CNTs-based samples showed higher compression strength compared to the pristine kaolin at low and moderate concentrations, none surpassed the strength of the PVA-only sample, indicating limitations in the reinforcing capabilities of CNTs in this context.

Interestingly, TiO_2_ nanofibers at low and moderate concentrations resulted in compression strengths higher than that of the PVA-only sample, highlighting the exceptional reinforcing capabilities of TiO_2_ nanofibers. However, at high concentrations, the compression strength of TiO_2_-based samples showed a slight decrease compared to the PVA-only sample, indicating a potential saturation effect or adverse interactions between TiO_2_ nanofibers and the kaolin matrix at higher concentrations.

3.2.2.2 **At 1050 °C:** The compression strength measurements conducted at a higher sintering temperature of 1050°C provide valuable insights into the mechanical behavior of the pristine and modified kaolin samples. These results reveal notable enhancements in compression strength compared to those observed at the lower sintering temperature of 900°C. The pristine kaolin exhibits a significant increase in compression strength, reaching 2.07 MPa at 1050°C, representing a notable improvement of 19.8% compared to the strength observed at 900°C. This enhancement can be attributed to the increased densification and crystallization of the kaolin matrix at higher temperatures, resulting in improved mechanical properties. Similarly, the compression strength of the PVA-modified kaolin sample also increases to 2.345 MPa at 1050°C, representing a considerable improvement of around 13% compared to the strength observed at 900°C. This increase is consistent with the densification and strengthening effects associated with higher sintering temperatures.

The SiO_2_ nanoparticle-modified kaolin samples exhibit remarkable improvements in compression strength upon increasing the sintering temperature from 900°C to 1050°C. At the highest SiO_2_ concentration (SiO2-3), the compression strength increases by approximately 53%, from 1.45 MPa at 900 °C to 2.18 MPa at 1050 °C. This substantial improvement underscores the synergistic effects of SiO_2_ nanoparticles and higher sintering temperatures in enhancing the mechanical properties of the kaolin matrix. Similarly, the moderate SiO_2_ concentration (SiO2-2) shows a significant increase in compression strength, with a 42.4% improvement from 1.56 MPa at 900°C to 2.25 MPa at 1050°C. This enhancement highlights the effectiveness of SiO_2_ nanoparticles in promoting densification and strengthening mechanisms at elevated temperatures. In contrast, the lowest SiO_2_ concentration (SiO2-1) exhibits a relatively modest improvement of only 13.3% in compression strength upon increasing the sintering temperature. This result suggests that the reinforcing effects of SiO_2_ nanoparticles may be less pronounced at lower concentrations, limiting the extent of improvement in mechanical properties [47].

Increasing the sintering temperature from 900 °C to 1050 °C resulted in varying degrees of improvement in compression strength across the AC-based samples. For the low AC concentration sample (AC-1), the compression strength significantly improved by 38.4% when the sintering temperature was increased to 1050°C. This enhancement can be attributed to the increased densification and crystallization of the kaolin matrix at higher temperatures, leading to improved mechanical properties. However, the magnitude of improvement in compression strength upon increasing the sintering temperature decreased with increasing AC content. Specifically, while the moderate concentration sample (AC-2) still exhibited a 14.7% improvement, further increases in AC content (AC-3) led to a decrease in compression strength at the higher sintering temperature. The observed decrease in compression strength for the AC-3 sample at 1050°C suggests that excessive AC content may have adverse effects on the structural integrity and mechanical properties of the kaolin matrix, potentially due to aggregation or inadequate dispersion of AC particles [48].

Comparing the results for CNTs-based samples sintered at 1050°C with those at 900°C, it is observed that increasing the sintering temperature from 900°C to 1050°C led to similar improvements in compression strength across all investigated concentrations of CNTs. Typically, the estimated improvement was around 19% with all concentrations. Accordingly, like 900 °C sintering temperature case, AC-1 and AC-2 samples revealed compression strength higher than of pristine kaolin while AC-3 still has lower value. All samples revealed lower compression strength compared to PVA sample. This consistent improvement suggests that the reinforcing effects of CNTs are not significantly influenced by variations in sintering temperature within the tested range. The consistent enhancement in compression strength observed at both sintering temperatures highlights the robustness and effectiveness of CNTs as reinforcing additives, particularly in promoting densification and strengthening mechanisms within the kaolin matrix.

At 900°C sintering temperature, both the low and moderate TiO_2_ nanofibers concentration samples (TiO2-1 and TiO2-2) exhibited compression strengths higher than that of the PVA-only sample, indicating the reinforcing effects of TiO_2_ nanofibers at these concentrations. However, when the sintering temperature was increased to 1050°C, only the TiO2-2 sample maintained its superiority in compression strength over the PVA sample, with a value of 2.47 MPa. In contrast, the compression strengths of the TiO2-1 and TiO2-3 samples were lower than that of the PVA sample at this temperature. Nonetheless, it is noteworthy that all three TiO_2_ nanofibers-based samples exhibited higher compression strengths compared to pristine kaolin, indicating the overall reinforcing effects of TiO_2_ nanofibers even at the higher sintering temperature.

The observed variations in compression strength among the TiO_2_ nanofibers-based samples can be attributed to several factors, including the morphological characteristics of TiO_2_ nanofibers, their interaction with the kaolin matrix, and the sintering conditions. At 1050°C, the higher sintering temperature promotes enhanced densification and crystallization of the kaolin matrix, potentially enhancing the reinforcing effects of TiO_2_ nanofibers.

**Fig 7:**
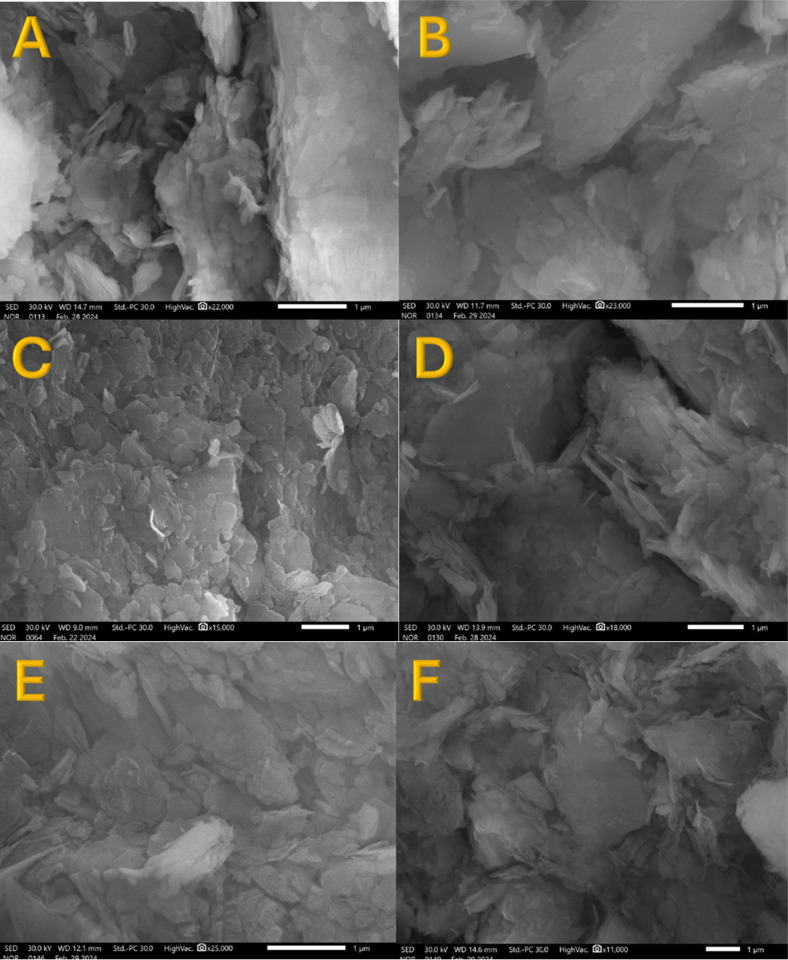
SEM images showing the microstructure of pristine kaolin. (A) and modified by PVA; (B), TiO2-1; (C), CNT-1; (D); SiO2-1; (E) and AC-3; (F) sintered at 1050 °C.

The mechanical properties measurement (Fig 6) revealed that the samples with higher compression strength compared to pristine kaolin are those modified by the addition of PVA and titanium oxide nanofibers. In contrast, other additives such as carbon nanotubes, silica nanoparticles, and activated carbon exhibited a slight positive effect or even a negative impact on the compression strength compared to pristine kaolin. To properly explain these findings, the microstructures of the sintered pristine and modified kaolin samples were studied using scanning electron microscopy (SEM) to image the cross-sections of the samples sintered at 1050°C (Fig 7).

In Fig 7A, the SEM image of pristine kaolin shows large particles, indicating good agglomeration of kaolin particles during the sintering process, which contributes to the improved compression strength of the prepared cube. However, several small non-agglomerated particles are also visible in the image. This incomplete agglomeration likely limits the overall mechanical integrity of the kaolin structure [49,50]. Fig 7B displays the SEM image of the sample modified by PVA only. The image shows large particles with very few free small particles, suggesting better agglomeration compared to pristine kaolin. The PVA acts as a binder, promoting particle cohesion and enhancing the overall structural integrity, which explains the observed increase in compression strength. The most notable improvement is observed with the addition of TiO_2_ nanofibers, as shown in Fig 7C. The SEM image reveals an almost solid surface with nearly all particles agglomerated and no discrete particles visible. The TiO_2_ nanofibers, due to their high surface area and long axial ratio, facilitate excellent particle bonding and create a dense, interconnected structure. This enhanced agglomeration and the formation of a more compact and uniform microstructure explain the highest compression strength observed for this sample. In contrast, the other additives (CNTs, SiO_2_ NPs, and activated carbon) did not achieve similar results. Fig 7D,7E, and 7F show SEM images of the kaolin samples modified by CNTs, SiO_2_, and AC, respectively. As shown, the size of the large particles is smaller compared to previous samples, and many non-agglomerated particles can be seen. This incomplete agglomeration indicates poor bonding and weaker structural integrity.

Although PVA was added with all additives and used alone, the highest compression strength was observed when this polymer was used alone and with TiO_2_ nanofibers. This difference in findings with SiO_2_, CNTs, and AC can be attributed to the specific interactions between the additives and the kaolin matrix. PVA alone provides a cohesive binder effect, improving agglomeration and strength. When combined with TiO_2_ nanofibers, the long axial ratio and high surface area of the nanofibers further enhance this effect, leading to superior mechanical properties. In contrast, SiO_2_ NPs, CNTs, and AC, despite being combined with PVA, did not facilitate the same level of particle bonding and agglomeration [51,52]. The smaller particle sizes and presence of non-agglomerated particles in the SEM images indicate that these additives may introduce more defects or porosity into the kaolin matrix. These defects can weaken the overall structure, leading to lower compression strength compared to the PVA-only and PVA-TiO_2_ samples.

**Fig 8:**
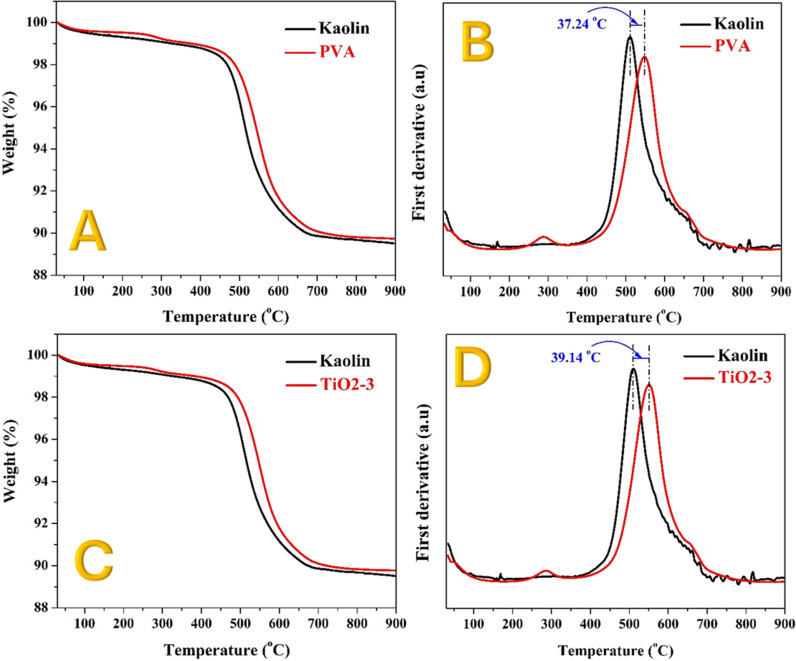
Thermal gravimetric analyses and the first derivative for the obtained data for the PVA; (A and B) and TiO_2_; (C and D) compared with the pristine kaolin.

**Fig 9:**
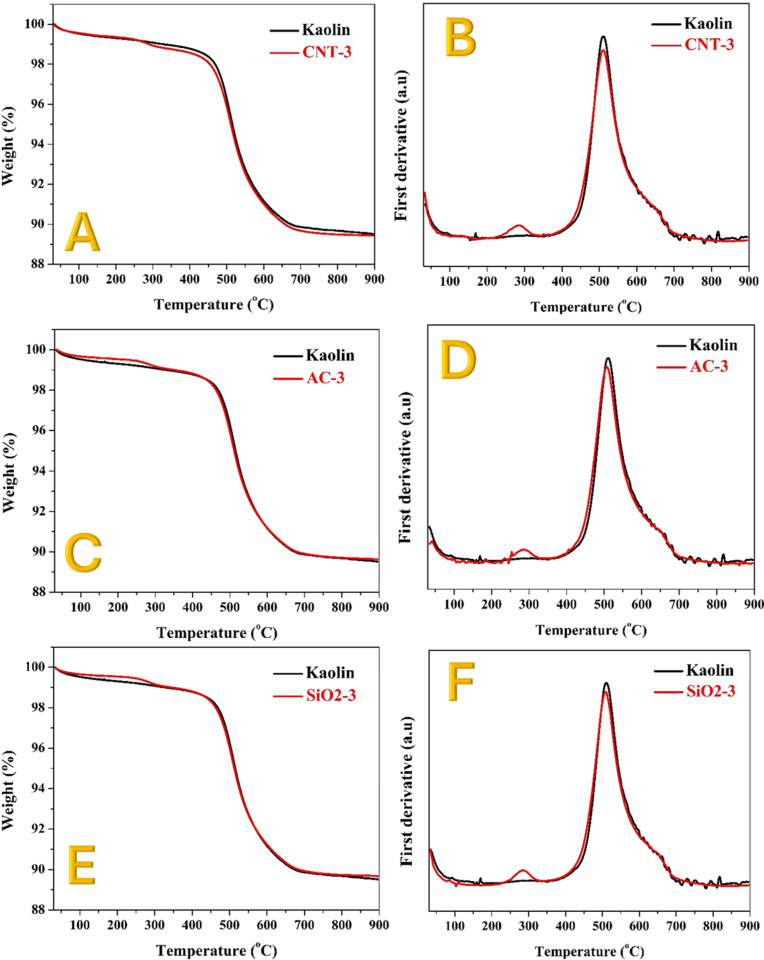
Thermal gravimetric analyses and the first derivative for the obtained data for the CNTs; (A and B), AC; (C and D) and SiO_2_; (E and F) compared with the pristine kaolin.

While SEM images provide qualitative insights into particle agglomeration and surface morphology, the absence of EDS elemental mapping limits precise phase identification. Future studies incorporating EDS and other spectroscopic techniques would enable a more detailed understanding of the microstructural and compositional evolution of these modified kaolin ceramics. However, to elucidate the underlying mechanisms behind the compression strength and SEM results, thermal gravimetric analysis (TGA) was performed on both pristine and modified kaolin samples. The TGA results, along with the first derivatives (Fig 8 and Fig 9), provide valuable insights into the sintering process and the effects of various additives on the thermal behavior of kaolin.

Fig 8A shows the TGA results for pristine and PVA-modified kaolin, while Fig 8B displays the first derivatives of the obtained data. A small peak is observed at around 290°C in all modified kaolin samples, which can be attributed to the decomposition of the polymer [53]. This peak indicates that the presence of PVA contributes to early-stage thermal events within the material, likely involving the degradation of organic components.

A more significant peak is observed at 511°C for pristine kaolin, which corresponds to a sharp increase in weight, indicating sintering reactions between kaolin particles. For the PVA-modified kaolin, this peak is shifted to 548°C, representing a positive shift of approximately 37°C. This shift suggests that the addition of PVA enhances the thermal stability of the kaolin particles, possibly by promoting better agglomeration and reducing the onset of sintering reactions.

Similar results are observed for the TiO_2_ nanofibers-modified kaolin (Fig 8C and 8D). The sintering reaction peak shifts slightly more, showing an increase of approximately 39°C. This further positive shift indicates that TiO_2_ nanofibers also contribute to the enhanced thermal stability of the kaolin matrix. The high surface area and long axial ratio of TiO_2_ nanofibers likely facilitate improved particle bonding and a more cohesive microstructure during sintering.

The TGA results and the corresponding first derivative calculations for the modified kaolin samples with SiO_2_ nanoparticles, CNTs, and AC are displayed in Fig 9. These results reveal that the addition of SiO_2_, CNTs, and AC does not significantly influence the sintering reaction peak compared to pristine kaolin, as no observable shift in the peak was detected. This lack of shift in the sintering reaction peak suggests that SiO_2_, CNTs, and AC do not enhance thermal stability or promote better particle agglomeration within the kaolin matrix. The sintering reaction peak for pristine kaolin appears at around 511°C, and this peak remains unchanged for the modified samples. This indicates that these additives do not alter the thermal behavior of kaolin during the sintering process.

Unlike TiO_2_ nanofibers, which have a high surface area and a long axial ratio that facilitates better particle bonding and thermal stability, SiO_2_ nanoparticles, CNTs, and AC may not possess the same properties. Their morphology and surface characteristics might not contribute to enhanced agglomeration or cohesion within the kaolin structure. Moreover, the inherent thermal stability of SiO_2_, CNTs, and AC might not be sufficient to influence the sintering reactions of kaolin. These materials may not provide the necessary thermal insulation or reinforcement to alter the thermal decomposition and reaction pathways of kaolin during sintering. The microstructural analysis from SEM images (Fig 7D, 7E, and 7F) supports these findings, showing that the kaolin samples modified with SiO_2_, CNTs, and AC contain many small, non-agglomerated particles. This lack of agglomeration likely leads to a more porous structure, which can negatively impact the mechanical properties and thermal behavior.

The TGA results align well with the compression strength measurements, providing a clear explanation for the observed mechanical properties. The positive shift in the sintering reaction peak for PVA and TiO_2_ nanofibers-modified samples indicates better thermal stability and improved particle agglomeration. This enhanced agglomeration leads to a denser and more interconnected structure, which directly translates to higher compression strength.

In summary, the improved thermal stability observed in PVA- and TiO_2_-modified kaolin samples can be attributed to the intrinsic properties of the additives and their interaction with the kaolin matrix. PVA, through hydrogen bonding and crosslinking, enhances thermal resistance by delaying chain mobility and degradation onset. TiO_2_ nanofibers, being thermally stable, serve as heat barriers and improve thermal resistance via uniform dispersion. In contrast, CNTs and AC, despite their inherent stability, exhibit weaker matrix interaction and potential oxidative behavior at elevated temperatures, limiting their stabilizing effects. SiO_2_ nanoparticles, while inert, offer minimal reinforcement due to limited interfacial bonding.

The results obtained in this study show a clear enhancement in the mechanical and thermal properties of kaolin ceramics when modified with PVA and TiO_2_ nanofibers. Compared to the unconfined compressive strength values reported in similar works, such as the study by Ghavami et al. [54], where the addition of 15% silica fume and 3% nano-silica increased the compressive strength of kaolinite clay by up to 70% and 55% respectively, the 2.47 MPa compressive strength achieved with TiO_2_ nanofibers in this study represents a notable improvement. Furthermore, our findings align with those of Bakr [9], who investigated phase transformations and densification behavior of Egyptian kaolin, yet our use of hybrid nanostructured additives demonstrates a more pronounced effect on sintering temperature shift and strength optimization. Despite such comparative evidence, it is important to note that the body of literature focusing specifically on nanostructure-enhanced kaolin ceramics remains limited. Most previous studies have addressed geotechnical stabilization or clayey soil reinforcement rather than high-temperature sintered ceramic applications. Therefore, this work contributes to filling that gap by targeting both mechanical and thermal performance enhancements relevant to refractory and structural ceramic industries.

#### 3.2.3 Apparent density.

**Fig 10:**
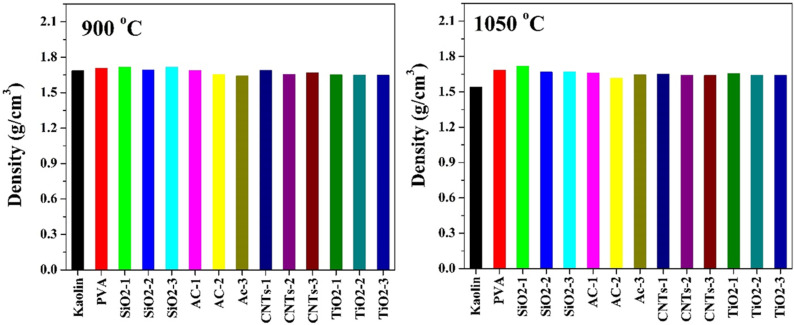
Apparent density of pristine and modified Egyptian kaolin sintered at two temperatures: 900 and 1050 °C.

The observed changes in the apparent density of the pristine and modified kaolin samples after sintering at different temperatures, in Fig 10, provide valuable insights into the effects of additives and sintering conditions on the densification behavior of the kaolin matrix.

At the lower sintering temperature of 900°C, minimal changes in the apparent density of the kaolin samples were observed, regardless of the additives used. This suggests that the densification of the kaolin matrix was limited at this temperature, resulting in negligible alterations in apparent density. However, upon increasing the sintering temperature to 1050°C, a significant decrease in the apparent density of the pristine kaolin was observed, with a reduction of approximately 9%. This decrease can be attributed to enhanced densification and consolidation of the kaolin particles at higher temperatures, leading to a reduction in pore volume and thus lower apparent density. The decrease in apparent density of the pristine kaolin at 1050°C can be also attributed to the increased diffusion and rearrangement of particles, leading to more efficient packing and reduced pore volume within the matrix.

Interestingly, for almost all additives investigated, including PVA, SiO2, AC, CNTs, and TiO2, no considerable decrease in apparent density was observed at the higher sintering temperature of 1050°C. This suggests that the additives did not significantly affect the densification behavior of the kaolin matrix under the tested conditions. The consistent apparent density values across different additive-modified samples at 1050°C indicate that the presence of additives did not impede the densification process, nor did they introduce additional porosity or voids within the kaolin matrix during sintering.

The lack of significant changes in apparent density with the addition of additives suggests that these materials did not hinder the densification process. Instead, they may have contributed to improved particle packing or promoted sintering mechanisms, such as grain boundary diffusion, resulting in denser microstructures [55]. Additionally, the interaction between additives and kaolin particles may have facilitated the formation of stronger interfacial bonds or secondary phases, which could have further contributed to densification without compromising apparent density [56].

**Fig 11:**
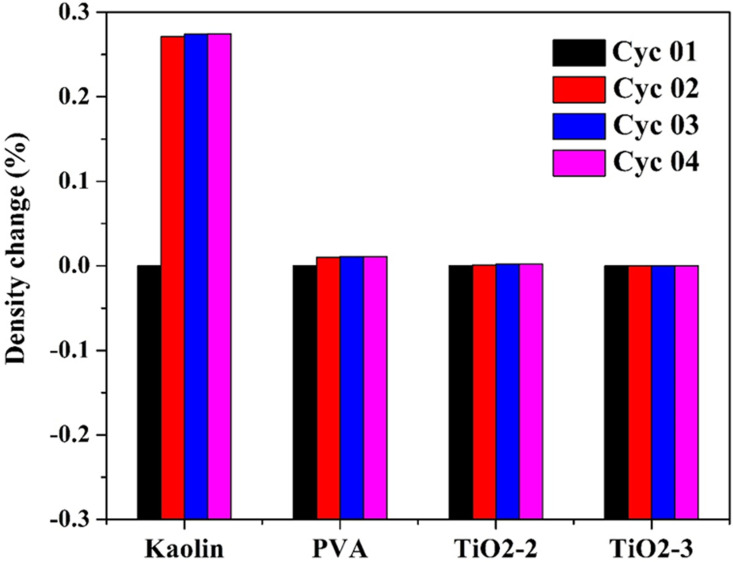
Percentage change in the apparent density of pristine and modified Egyptian kaolin sintered at 1050 °C for 4 successive cycles.

The results shown in Fig 11 reveal the percentage change in the apparent density of pristine and modified kaolin samples after being heated to 1050°C for four successive cycles. The pristine kaolin showed a slight increase in apparent density by 0.27% after the first cycle, followed by negligible changes in the subsequent cycles. In contrast, the PVA-modified sample exhibited a minimal increase of 0.011% in apparent density after the first cycle, and the TiO_2_ nanofibers-modified kaolin displayed no change in apparent density throughout all cycles.

The initial increase in the apparent density of pristine kaolin suggests some degree of particle rearrangement and further densification during the first heating cycle. This phenomenon is typical in ceramic materials as initial heating can cause slight shrinkage and compaction. The subsequent stability in density indicates that the kaolin structure reaches a relatively stable state after the initial cycle, with minimal further densification or rearrangement.

The insignificant increase in density (0.011%) after the first cycle suggests that PVA aids in achieving better initial compaction and particle bonding during sintering. This could be attributed to the polymer’s ability to act as a binder, enhancing the cohesion between kaolin particles. The stability in density across successive cycles indicates that the PVA-modified kaolin retains its structural integrity and resists further changes upon repeated heating.

The absence of any change in the apparent density over all four cycles is particularly noteworthy. This result suggests that TiO_2_ NFs provide exceptional thermal stability and structural reinforcement to the kaolin matrix. The incorporation of TiO_2_ NFs likely creates a robust network within the kaolin, minimizing particle movement and densification during repeated heating cycles. The findings highlight the significant potential of TiO_2_ NFs-modified kaolin as a refractory material, particularly in kiln applications where materials are subjected to repeated thermal cycling.

The unchanged apparent density of TiO_2_ NFs-modified kaolin over multiple heating cycles indicates superior thermal stability. This property is crucial for refractory materials that must maintain structural integrity and mechanical strength under fluctuating high-temperature conditions. This reinforcement minimizes microstructural changes, preventing deterioration and extending the lifespan of refractory components.

#### 3.2.4 Water absorption.

The water absorption characteristic of ceramic materials significantly impacts their performance, durability, chemical resistance, thermal properties, aesthetics, and environmental sustainability. Understanding and controlling water absorption is essential in designing ceramic products tailored to specific applications, ensuring optimal performance, longevity, and suitability for diverse environments and functional requirements.

**Fig 12:**
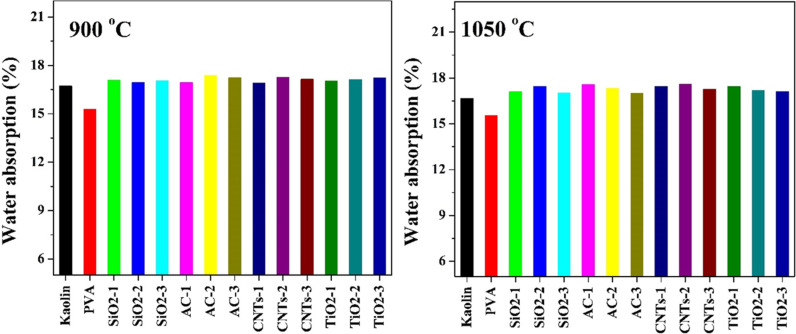
Water absorption of pristine and modified Egyptian kaolin sintered at two temperatures: 900 and 1050 °C.

The water absorption results depicted in Fig 12 provide valuable insights into the effects of additives and sintering temperature on the porosity and moisture uptake behavior of kaolin-based ceramics. The addition of PVA resulted in a significant reduction in water absorption for both sintering temperatures (900°C and 1050°C), indicating improved densification and reduced porosity in the kaolin matrix. PVA, being a polymeric binder, can act as a sintering aid and promote particle packing, leading to enhanced compaction and reduced pore volume during firing. The observed decrease in water absorption with PVA addition highlights its effectiveness in improving the impermeability and moisture resistance of the ceramic material, making it suitable for applications requiring low water absorption, such as in sanitaryware or tiles.

In contrast to PVA, the other additives used in conjunction showed minimal impact on water absorption, particularly at 900°C sintering temperature. This trivial effect on water absorption suggests that these additives, including SiO_2_, AC, CNTs, and TiO_2_, may not significantly alter the microstructure or porosity of the kaolin matrix under the tested conditions. The lack of substantial reduction in water absorption with these additives may be attributed to factors such as inadequate dispersion, weak interaction with the kaolin particles, or limited sintering promotion capabilities compared to PVA.

The slight increase in water absorption observed for some additives at 1050°C, such as SiO2-2, AC-1, CNTs-1, CNTs-2, and TiO2-1, may be attributed to factors such as phase transformations, secondary phase formation, or incomplete densification at higher temperatures.

Interestingly, increasing the sintering temperature from 900°C to 1050°C did not lead to a significant change in water absorption for all samples, indicating that the effects of sintering temperature on porosity reduction were limited. This observation suggests that the sintering conditions employed may have already achieved near-optimal densification at 900°C, with further increases in temperature providing marginal improvements in pore closure or compaction.

The findings underscore the importance of PVA as an effective additive for reducing water absorption in kaolin-based ceramics, highlighting its potential for enhancing moisture resistance and durability in various applications.

#### 3.2.5 Porosity.

**Fig 13:**
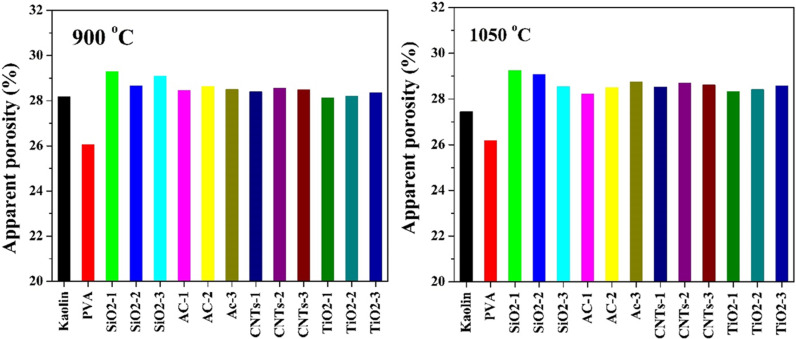
Porosity of pristine and modified Egyptian kaolin sintered at two temperatures: 900 and 1050 °C.

The porosity results presented in Fig 13 provide valuable insights into the effects of additives and sintering temperature on the pore structure and density of kaolin-based ceramics. These findings complement the water absorption results discussed earlier, highlighting the relationship between porosity and water absorption characteristics.

Consistent with the water absorption findings, addition of PVA led to a significant reduction in porosity for both sintering temperatures (900°C and 1050°C). This reduction in porosity corresponds to the decreased water absorption observed in the PVA-modified samples. The denser microstructure achieved with PVA contributes to lower water absorption due to reduced accessibility of water into the pores.

In contrast to PVA, the other additives used alongside showed minimal influence on porosity reduction, particularly evident at 900°C sintering temperature. This finding aligns with the trivial impact of these additives on water absorption, indicating a lack of significant alteration in pore structure. The minimal changes in porosity with these additives suggest that their effects on water absorption are closely linked to the density and distribution of pores within the ceramic matrix.

At 1050°C sintering temperature, most additives exhibited an opposite influence on porosity compared to PVA, resulting in increased porosity relative to pristine kaolin. This corresponds to the observed increase in water absorption with certain additives at this temperature. The increase in porosity with these additives may be attributed to factors such as phase transformations, secondary phase formation, or incomplete densification, which could introduce additional pores or defects within the ceramic matrix.

Increasing the sintering temperature from 900°C to 1050°C did not significantly alter the porosity of the samples. This observation is consistent with the minimal changes in water absorption across different sintering temperatures, indicating limited effects on pore closure or compaction.

The additives used in this study (PVA, TiO_2_ nanofibers, SiO_2_ nanoparticles, carbon nanotubes, and activated carbon) are commercially available and can be sourced at relatively low costs when purchased in bulk. This makes the approach economically feasible for industrial applications. Moreover, the processing methods employed in this study, such as mixing, drying, and sintering, are well-established industrial techniques. These methods are routinely used in ceramic manufacturing and can be easily scaled up for mass production without requiring specialized equipment or highly complex procedures. The enhancement of mechanical properties, particularly compression strength and reduced water absorption, achieved with the addition of these materials can lead to longer lasting and more durable ceramic products. This, in turn, can reduce maintenance and replacement costs in various applications, offering significant economic benefits over time. The significant improvements observed with PVA and TiO_2_ nanofibers suggest that even small additions of these materials can result in substantial performance gains, making the approach cost-efficient.

Table 3 summarizes the influence of each additive on the investigated physical and mechanical properties of the Egyptian Kaolin.

**Table 3:** Effect of each additive on Kaolin ceramic properties at different sintering temperatures.

Additive	Sintering Temp.	Plasticity	Compressive Strength	Porosity	Water Absorption	Density	Thermal Stability
**PVA**	900 °C	**↑↑**	**↑**	**↓**	**↓**	**↑**	**↑**
	1050 °C		**↑↑**	**↓↓**	**↓↓**	**↑↑**	**↑↑**
**TiO**_**2**_ **NFs**	900 °C	**↑**	**↑**	**↓**	**↓**	**↑**	**↑**
	1050 °C		**↑↑**	**↓**	**↓**	**↑↑**	**↑↑**
**CNTs**	900 °C	**↓**	**↑**	**↑**	**↑**	**↓**	**~**
	1050 °C		**~**	**↑**	**↑**	**↓**	**~**
**SiO**_**2**_ **NPs**	900 °C	**~**	**~**	**~**	**~**	**~**	**↑**
	1050 °C		**~**	**~**	**~**	**~**	**↑**
**AC**	900 °C	**↓**	**~**	**↑**	**↑**	**↓**	**↓**
	1050 °C		**~**	**↑**	**↑**	**↓**	**↓**

↑↑: Significant increase ↑ : Moderate increase ~ : No major change ↓ : Moderate decrease ↓↓: Significant decrease

The observed improvements in the mechanical and thermal properties of the modified kaolin ceramics can be attributed to the distinct interactions between the additives and the kaolin matrix during processing and sintering. Polyvinyl alcohol (PVA), as a water-soluble polymer, enhances plasticity during shaping and forms hydrogen bonds with kaolin particles, promoting tighter packing and reducing microcracks upon drying. Upon pyrolysis, PVA leaves a residual carbon phase that can contribute to densification and microstructural coherence. TiO_2_ nanofibers, due to their high thermal stability and fibrous morphology, act as structural bridges within the matrix, facilitating stress distribution and inhibiting crack propagation. The uniform dispersion of TiO_2_ also promotes localized sintering necks between kaolin particles, enhancing compressive strength. In contrast, CNTs and AC, despite having excellent intrinsic properties, tend to agglomerate and display weak interfacial bonding with the hydrophilic kaolin, which may explain their limited reinforcement effect. Surface acid treatment was applied to mitigate this, but full compatibility may not have been achieved. SiO_2_ NPs, being inert and spherical, contribute minimally to mechanical bonding but may assist in particle rearrangement during sintering. These varied mechanisms highlight the importance of interfacial chemistry, morphology, and thermal behavior in designing optimized kaolin–additive composites.

Overall, the enhancements in compressive strength and thermal stability observed in this study make the modified kaolin ceramics suitable for demanding industrial applications, such as refractory linings in high-temperature furnaces, where mechanical integrity and heat resistance are critical. Additionally, the reduction in porosity and water absorption supports the use of these materials in construction tiles and membrane supports in filtration systems. The use of cost-effective and scalable additives like PVA and TiO_2_ nanofibers further supports the potential for widespread industrial adoption.

## 4. Conclusions

This study elucidated novel strategies for enhancing Egyptian kaolin-based ceramics through additive engineering and optimization of sintering conditions. The incorporation of various nanostructural materials, including poly(vinyl alcohol) (PVA), silica nanoparticles (SiO_2_), activated carbon (AC), carbon nanotubes (CNTs), and titanium oxide nanofibers (TiO_2_), significantly influenced the properties of the ceramics. Comprehensive characterization revealed improvements in plasticity, compression strength, apparent density, water absorption, and porosity. Notably, PVA addition resulted in notable enhancements in plasticity and compression strength, while TiO_2_ sample exhibited the highest compression strength at 2.47 MPa. The findings underscore the efficacy of additive engineering in tailoring the properties of Egyptian kaolin ceramics, with potential applications across diverse industries. Further research exploring the synergistic effects of additives and refining sintering parameters could lead to the development of high-performance ceramic materials with enhanced functionality and durability.

While the current study demonstrates significant improvements in the mechanical and thermal properties of kaolin ceramics using nanostructured additives, several areas warrant further investigation. Future work should incorporate more detailed chemical characterization techniques such as EDS elemental mapping or XPS to confirm phase distribution and surface interactions. Additionally, optimizing the concentration of additives and exploring synergistic effects between multiple nanostructures may further enhance performance. Long-term durability testing and scale-up trials under real industrial conditions are also essential to validate these materials for practical applications.
